# Crystal structure of di-μ_2_-chlorido-bis­[(1-aza-4-azoniabi­cyclo­[2.2.2]octane-κ*N* ^1^)di­chlorido­dicadmium]

**DOI:** 10.1107/S2056989015023361

**Published:** 2015-12-12

**Authors:** Jing-Jing Yan, Qi-Jian Pan, Li-Zhuang Chen

**Affiliations:** a School of Environmental and Chemical Engineering, Jiangsu University of Science and Technology, Zhenjiang 212003, People’s Republic of China

**Keywords:** crystal structure, cadmium, DABCO, hydrogen bonding

## Abstract

In the structure of the binuclear title compound, [Cd_2_(C_6_H_13_N_2_)_2_Cl_6_], two Cd^II^ atoms are bridged by two Cl^−^ ligands, defining a centrosymmetric Cd_2_Cl_2_ motif. Each metal cation is additionally coordinated by two Cl^−^ ligands and the N atom of a protonated 1,4-di­aza­bicyclo­[2.2.2]octane (H-DABCO)^+^ ligand, leading to an overall trigonal–bipyramidal coordination environment with one of the bridging Cl^−^ ligands and the N atom at the apical sites. In the crystal, the neutral dimers are linked *via* N—H⋯Cl hydrogen bonds, forming a two-dimensional network expanding parallel to (100).

## Related literature   

For a study on phase transition of related Cd_2_(DABCO-CH_2_Cl)_2_(μ-Cl_2_), see: Chen *et al.* (2014 ▸). Mononuclear and dinuclear bromide-nitrite cadmium complexes with DABCO derivatives were reported by Cai (2011 ▸).
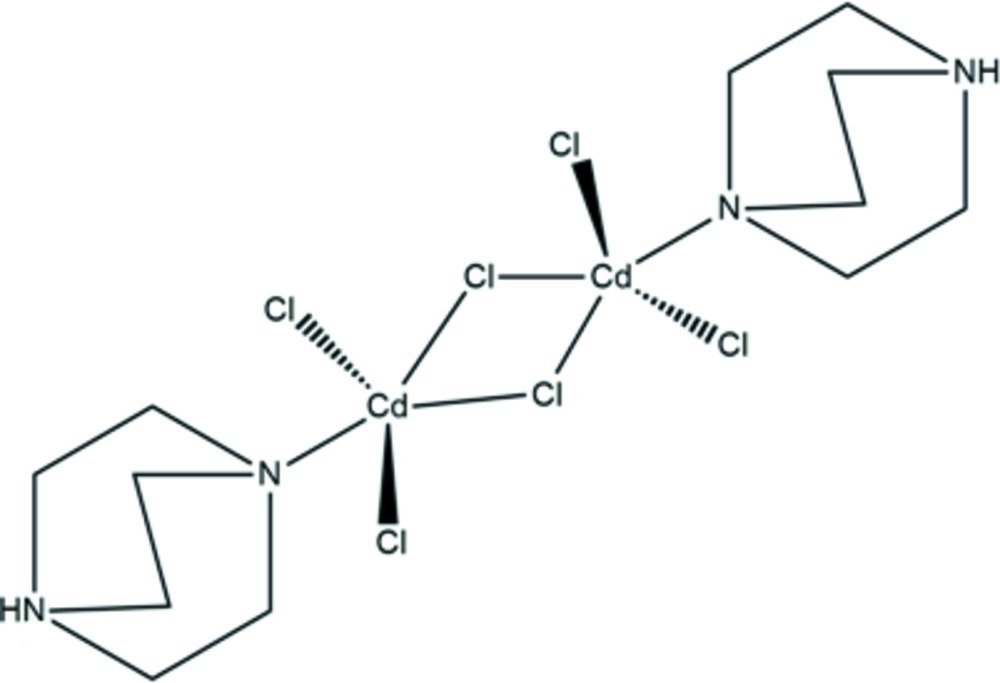


## Experimental   

### Crystal data  

[Cd_2_(C_6_H_13_N_2_)_2_Cl_6_]
*M* *_r_* = 663.86
Orthorhombic, 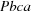

*a* = 12.317 (2) Å
*b* = 12.289 (2) Å
*c* = 14.440 (2) Å
*V* = 2185.7 (6) Å^3^
*Z* = 4
Mo *K*α radiation
μ = 2.68 mm^−1^
*T* = 296 K
0.3 × 0.2 × 0.2 mm

### Data collection  

Bruker APEXII CCD diffractometer
Absorption correction: multi-scan (*SADABS*; Bruker, 2004 ▸) *T*_min_ = 0.500, *T*_max_ = 0.616
14939 measured reflections
1924 independent reflections
1752 reflections with *I* > 2σ(*I*)
*R*_int_ = 0.025

### Refinement  

*R*[*F* ^2^ > 2σ(*F* ^2^)] = 0.054
*wR*(*F* ^2^) = 0.183
*S* = 1.12
1924 reflections
109 parameters
30 restraints
H-atom parameters constrained
Δρ_max_ = 1.98 e Å^−3^
Δρ_min_ = −1.65 e Å^−3^

### 

Data collection: *APEX2* (Bruker, 2004 ▸); cell refinement: *SAINT* (Bruker, 2004 ▸); data reduction: *SAINT*; program(s) used to solve structure: *SHELXS97* (Sheldrick, 2008 ▸); program(s) used to refine structure: *SHELXL97* (Sheldrick, 2008 ▸); molecular graphics: *DIAMOND* (Brandenburg, 2006 ▸); software used to prepare material for publication: *OLEX2* (Dolomanov *et al.*, 2009 ▸).

## Supplementary Material

Crystal structure: contains datablock(s) I. DOI: 10.1107/S2056989015023361/wm5244sup1.cif

**Fig. 1.**
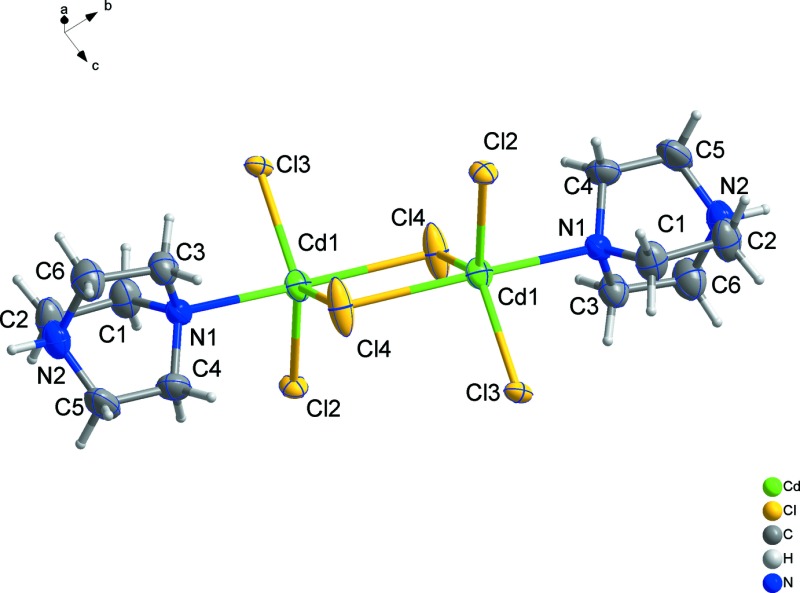
The molecular structure of the dinuclear complex in the title compound. Displacement ellipsoids are drawn at the 30% probability level. The left part of the binuclear complex is generated by symmetry code −x + 1, −y, −z + 1.

Structure factors: contains datablock(s) I. DOI: 10.1107/S2056989015023361/wm5244Isup2.hkl

x y z . DOI: 10.1107/S2056989015023361/wm5244fig1.tif
The mol­ecular structure of the dinuclear complex in the title compound. Displacement ellipsoids are drawn at the 30% probability level. The left part of the binuclear complex is generated by symmetry code −*x* + 1, −*y*, −*z* + 1.

**Fig. 2.**
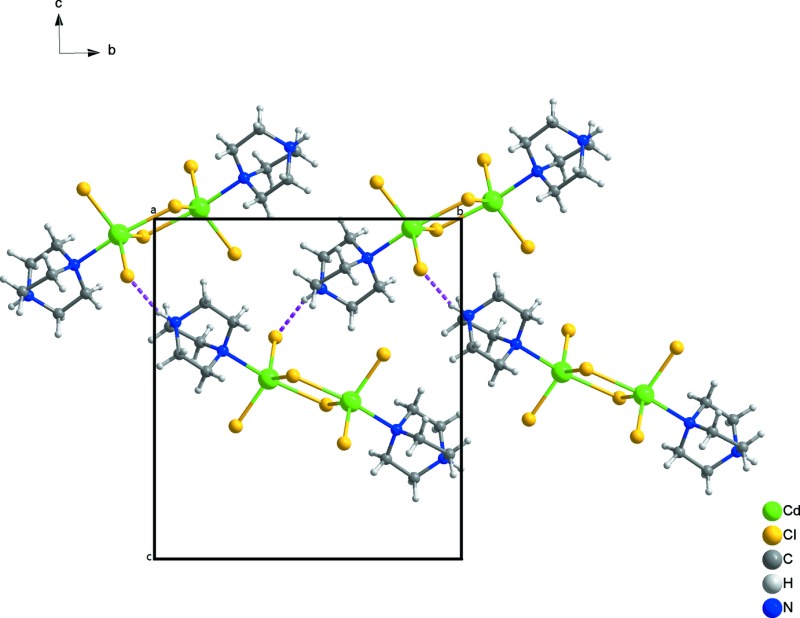
View onto a layer of complexes in the title compound with N—H···Cl hydrogen bonds drawn as dashed lines.

. DOI: 10.1107/S2056989015023361/wm5244fig2.tif
View onto a layer of complexes in the title compound with N—H⋯Cl hydrogen bonds drawn as dashed lines.

CCDC reference: 1440782

Additional supporting information:  crystallographic information; 3D view; checkCIF report

## Figures and Tables

**Table 1:** Hydrogen-bond geometry (Å, °)

*D*—H⋯*A*	*D*—H	H⋯*A*	*D*⋯*A*	*D*—H⋯*A*
N2—H2⋯Cl3^i^	0.91	2.33	3.205 (3)	162

Symmetry code: (i) 
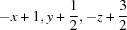
.

## supplementary crystallographic information

### S1. Synthesis and crystallization

CdCl_2_·2.5H_2_O (2.28 g, 10 mmol) and 1,4-di­aza­bicyclo [2.2.2]o­ctan (1.12 g, 10 mmol) were mixed in water (20 ml). After being stirred for 30 min, the reaction mixture was filtered and evaporated slowly at room temperature for 3 days. Colourless block-like crystals were obtained.

### S2. Refinement

C-bound H atoms were placed in geometrically idealized positions and constrained to ride on their parent atoms, with C—H = 0.97 Å and *U*_iso_(H) = 1.2*U*_eq_(C). The H atom of the protonated N2 atom was discernible from a difference map. It was modelled with N—H = 0.91 Å and *U*_iso_(H) = 1.2*U*_eq_(N). The maximum and minimum electron density peaks are found 0.20 Å from atom Cl3 and 0.27 Å from atom Cd1, respectively.

### Crystal data

**Table:**

[Cd_2_(C_6_H_13_N_2_)_2_Cl_6_]	*D*_x_ = 2.017 Mg m^−^^3^
*M**_r_* = 663.86	Mo *K*α radiation, λ = 0.71073 Å
Orthorhombic, *P**b**c**a*	Cell parameters from 6044 reflections
*a* = 12.317 (2) Å	θ = 2.7–27.4°
*b* = 12.289 (2) Å	µ = 2.68 mm^−^^1^
*c* = 14.440 (2) Å	*T* = 296 K
*V* = 2185.7 (6) Å^3^	Block, colorless
*Z* = 4	0.3 × 0.2 × 0.2 mm
*F*(000) = 1296	

### Data collection

**Table:**

Bruker APEXII CCD diffractometer	1924 independent reflections
Radiation source: fine-focus sealed tube	1752 reflections with *I* > 2σ(*I*)
Graphite monochromator	*R*_int_ = 0.025
phi and ω scans	θ_max_ = 25.0°, θ_min_ = 2.7°
Absorption correction: multi-scan (*SADABS*; Bruker, 2004)	*h* = −13→14
*T*_min_ = 0.500, *T*_max_ = 0.616	*k* = −14→14
14939 measured reflections	*l* = −17→16

### Refinement

**Table:**

Refinement on *F*^2^	Primary atom site location: structure-invariant direct methods
Least-squares matrix: full	Secondary atom site location: difference Fourier map
*R*[*F*^2^ > 2σ(*F*^2^)] = 0.054	Hydrogen site location: inferred from neighbouring sites
*wR*(*F*^2^) = 0.183	H-atom parameters constrained
*S* = 1.12	*w* = 1/[σ^2^(*F*_o_^2^) + (0.1089*P*)^2^ + 19.3777*P*] where *P* = (*F*_o_^2^ + 2*F*_c_^2^)/3
1924 reflections	(Δ/σ)_max_ = 0.006
109 parameters	Δρ_max_ = 1.98 e Å^−^^3^
30 restraints	Δρ_min_ = −1.65 e Å^−^^3^

### Special details

**Table:**

Geometry. All e.s.d.'s (except the e.s.d. in the dihedral angle between two l.s. planes) are estimated using the full covariance matrix. The cell e.s.d.'s are taken into account individually in the estimation of e.s.d.'s in distances, angles and torsion angles; correlations between e.s.d.'s in cell parameters are only used when they are defined by crystal symmetry. An approximate (isotropic) treatment of cell e.s.d.'s is used for estimating e.s.d.'s involving l.s. planes.
Refinement. Refinement of *F*^2^ against ALL reflections. The weighted *R*-factor *wR* and goodness of fit *S* are based on *F*^2^, conventional *R*-factors *R* are based on *F*, with *F* set to zero for negative *F*^2^. The threshold expression of *F*^2^ > σ(*F*^2^) is used only for calculating *R*-factors(gt) *etc*. and is not relevant to the choice of reflections for refinement. *R*-factors based on *F*^2^ are statistically about twice as large as those based on *F*, and *R*- factors based on ALL data will be even larger.

### Fractional atomic coordinates and isotropic or equivalent isotropic displacement parameters (Å^2^)

**Table:**

	*x*	*y*	*z*	*U*_iso_*/*U*_eq_	
Cd1	0.42960 (2)	0.138551 (19)	0.535783 (17)	0.03153 (7)	
Cl2	0.37788 (6)	0.25484 (6)	0.40100 (5)	0.02553 (17)	
Cl3	0.28196 (6)	0.11273 (6)	0.65621 (5)	0.02369 (17)	
Cl4	0.61656 (7)	0.05552 (8)	0.54294 (8)	0.0606 (3)	
C1	0.4234 (3)	0.3697 (3)	0.6469 (3)	0.0453 (12)	
H1A	0.3732	0.3344	0.6890	0.054*	
H1B	0.3833	0.3921	0.5923	0.054*	
C3	0.5670 (3)	0.2551 (3)	0.7038 (2)	0.0401 (9)	
H3A	0.6263	0.2078	0.6853	0.048*	
H3B	0.5177	0.2128	0.7418	0.048*	
N1	0.5090 (2)	0.2930 (2)	0.62028 (18)	0.0284 (7)	
C4	0.5890 (3)	0.3502 (3)	0.5617 (3)	0.0418 (10)	
H4A	0.5526	0.3799	0.5078	0.050*	
H4B	0.6432	0.2987	0.5404	0.050*	
C2	0.4742 (3)	0.4710 (3)	0.6946 (3)	0.0512 (12)	
H2A	0.4624	0.5352	0.6568	0.061*	
H2B	0.4403	0.4829	0.7544	0.061*	
N2	0.5912 (3)	0.4521 (3)	0.7064 (2)	0.0429 (8)	
H2	0.6208	0.5096	0.7370	0.051*	
C5	0.6446 (4)	0.4414 (3)	0.6140 (3)	0.0553 (12)	
H5A	0.7211	0.4251	0.6218	0.066*	
H5B	0.6381	0.5091	0.5799	0.066*	
C6	0.6123 (4)	0.3495 (3)	0.7611 (3)	0.0537 (10)	
H6A	0.5763	0.3531	0.8208	0.064*	
H6B	0.6895	0.3400	0.7713	0.064*	

### Atomic displacement parameters (Å^2^)

**Table:**

	*U*^11^	*U*^22^	*U*^33^	*U*^12^	*U*^13^	*U*^23^
Cd1	0.03132 (14)	0.02795 (13)	0.03533 (14)	0.00383 (9)	−0.00315 (9)	−0.00071 (9)
Cl2	0.0293 (3)	0.0249 (3)	0.0224 (3)	0.0010 (3)	−0.0047 (3)	0.0066 (3)
Cl3	0.0201 (3)	0.0231 (3)	0.0278 (3)	0.0002 (3)	0.0055 (3)	0.0068 (3)
Cl4	0.0362 (4)	0.0375 (4)	0.1082 (7)	0.0139 (4)	−0.0311 (4)	−0.0347 (4)
C1	0.0315 (19)	0.042 (2)	0.062 (2)	0.0035 (15)	0.0005 (17)	−0.0087 (17)
C3	0.0468 (17)	0.0325 (15)	0.0408 (16)	0.0022 (13)	−0.0054 (14)	0.0003 (14)
N1	0.0269 (12)	0.0237 (12)	0.0346 (13)	−0.0007 (10)	0.0030 (11)	0.0011 (11)
C4	0.0393 (18)	0.0414 (19)	0.0448 (19)	−0.0063 (16)	0.0128 (17)	0.0046 (16)
C2	0.041 (2)	0.0406 (19)	0.072 (2)	0.0050 (17)	0.003 (2)	−0.0175 (19)
N2	0.0411 (14)	0.0361 (14)	0.0515 (15)	−0.0060 (12)	−0.0063 (13)	−0.0092 (12)
C5	0.054 (2)	0.0381 (19)	0.074 (3)	−0.0152 (17)	0.024 (2)	0.0006 (19)
C6	0.0584 (17)	0.0475 (16)	0.0551 (17)	−0.0017 (15)	−0.0138 (16)	−0.0050 (15)

### Geometric parameters (Å, º)

**Table:**

Cd1—Cl2	2.4972 (8)	N1—C4	1.477 (5)
Cd1—Cl3	2.5361 (8)	C4—H4A	0.9700
Cd1—Cl4^i^	2.7025 (11)	C4—H4B	0.9700
Cd1—Cl4	2.5207 (10)	C4—C5	1.515 (6)
Cd1—N1	2.460 (3)	C2—H2A	0.9700
Cl4—Cd1^i^	2.7025 (11)	C2—H2B	0.9700
C1—H1A	0.9700	C2—N2	1.470 (5)
C1—H1B	0.9700	N2—H2	0.9100
C1—N1	1.465 (5)	N2—C5	1.493 (5)
C1—C2	1.555 (6)	N2—C6	1.510 (5)
C3—H3A	0.9700	C5—H5A	0.9700
C3—H3B	0.9700	C5—H5B	0.9700
C3—N1	1.477 (4)	C6—H6A	0.9700
C3—C6	1.530 (6)	C6—H6B	0.9700
			
Cl2—Cd1—Cl3	115.03 (3)	N1—C4—H4B	109.3
Cl2—Cd1—Cl4	119.78 (3)	N1—C4—C5	111.6 (3)
Cl2—Cd1—Cl4^i^	97.09 (3)	H4A—C4—H4B	108.0
Cl3—Cd1—Cl4^i^	91.56 (3)	C5—C4—H4A	109.3
Cl4—Cd1—Cl3	125.19 (3)	C5—C4—H4B	109.3
Cl4—Cd1—Cl4^i^	81.50 (3)	C1—C2—H2A	110.0
N1—Cd1—Cl2	92.66 (6)	C1—C2—H2B	110.0
N1—Cd1—Cl3	92.39 (6)	H2A—C2—H2B	108.3
N1—Cd1—Cl4	85.91 (7)	N2—C2—C1	108.6 (3)
N1—Cd1—Cl4^i^	166.77 (6)	N2—C2—H2A	110.0
Cd1—Cl4—Cd1^i^	98.50 (3)	N2—C2—H2B	110.0
H1A—C1—H1B	108.2	C2—N2—H2	109.0
N1—C1—H1A	109.7	C2—N2—C5	110.0 (3)
N1—C1—H1B	109.7	C2—N2—C6	111.2 (3)
N1—C1—C2	110.0 (3)	C5—N2—H2	109.0
C2—C1—H1A	109.7	C5—N2—C6	108.5 (3)
C2—C1—H1B	109.7	C6—N2—H2	109.0
H3A—C3—H3B	107.9	C4—C5—H5A	110.1
N1—C3—H3A	109.2	C4—C5—H5B	110.1
N1—C3—H3B	109.2	N2—C5—C4	108.2 (3)
N1—C3—C6	112.2 (3)	N2—C5—H5A	110.1
C6—C3—H3A	109.2	N2—C5—H5B	110.1
C6—C3—H3B	109.2	H5A—C5—H5B	108.4
C1—N1—Cd1	109.9 (2)	C3—C6—H6A	110.4
C1—N1—C3	109.7 (3)	C3—C6—H6B	110.4
C1—N1—C4	108.9 (3)	N2—C6—C3	106.7 (3)
C3—N1—Cd1	110.70 (19)	N2—C6—H6A	110.4
C4—N1—Cd1	110.4 (2)	N2—C6—H6B	110.4
C4—N1—C3	107.2 (3)	H6A—C6—H6B	108.6
N1—C4—H4A	109.3		
			
Cd1—N1—C4—C5	−176.4 (2)	C1—C2—N2—C5	63.5 (4)
Cl2—Cd1—Cl4—Cd1^i^	−93.35 (4)	C1—C2—N2—C6	−56.8 (4)
Cl2—Cd1—N1—C1	67.3 (2)	C3—N1—C4—C5	−55.7 (4)
Cl2—Cd1—N1—C3	−171.4 (2)	N1—Cd1—Cl4—Cd1^i^	175.92 (7)
Cl2—Cd1—N1—C4	−52.9 (2)	N1—C1—C2—N2	−5.9 (5)
Cl3—Cd1—Cl4—Cd1^i^	85.88 (4)	N1—C3—C6—N2	−5.6 (4)
Cl3—Cd1—N1—C1	−47.9 (2)	N1—C4—C5—N2	−6.1 (4)
Cl3—Cd1—N1—C3	73.4 (2)	C2—C1—N1—Cd1	−176.3 (3)
Cl3—Cd1—N1—C4	−168.1 (2)	C2—C1—N1—C3	61.8 (4)
Cl4^i^—Cd1—Cl4—Cd1^i^	0.0	C2—C1—N1—C4	−55.2 (4)
Cl4—Cd1—N1—C1	−173.0 (2)	C2—N2—C5—C4	−56.7 (4)
Cl4^i^—Cd1—N1—C1	−155.2 (3)	C2—N2—C6—C3	63.1 (4)
Cl4—Cd1—N1—C3	−51.7 (2)	C5—N2—C6—C3	−58.0 (4)
Cl4^i^—Cd1—N1—C3	−33.9 (4)	C6—C3—N1—Cd1	−176.8 (3)
Cl4—Cd1—N1—C4	66.8 (2)	C6—C3—N1—C1	−55.5 (4)
Cl4^i^—Cd1—N1—C4	84.7 (4)	C6—C3—N1—C4	62.7 (4)
C1—N1—C4—C5	62.9 (4)	C6—N2—C5—C4	65.1 (4)

Symmetry code: (i) −*x*+1, −*y*, −*z*+1.

### Hydrogen-bond geometry (Å, º)

**Table:**

*D*—H···*A*	*D*—H	H···*A*	*D*···*A*	*D*—H···*A*
N2—H2···Cl3^ii^	0.91	2.33	3.205 (3)	162

Symmetry code: (ii) −*x*+1, *y*+1/2, −*z*+3/2.
