# Supplementary figures and images for: Crystal structure of di-μ2-chlorido-bis­[(1-aza-4-azoniabi­cyclo­[2.2.2]octane-κN 1)di­chlorido­dicadmium]
Source: Acta Crystallogr E Crystallogr Commun. 2015 Dec 12;71(Pt 12):m259–60. doi: 10.1107/S2056989015023361 (PMC4719863; doi:10.1107/S2056989015023361)

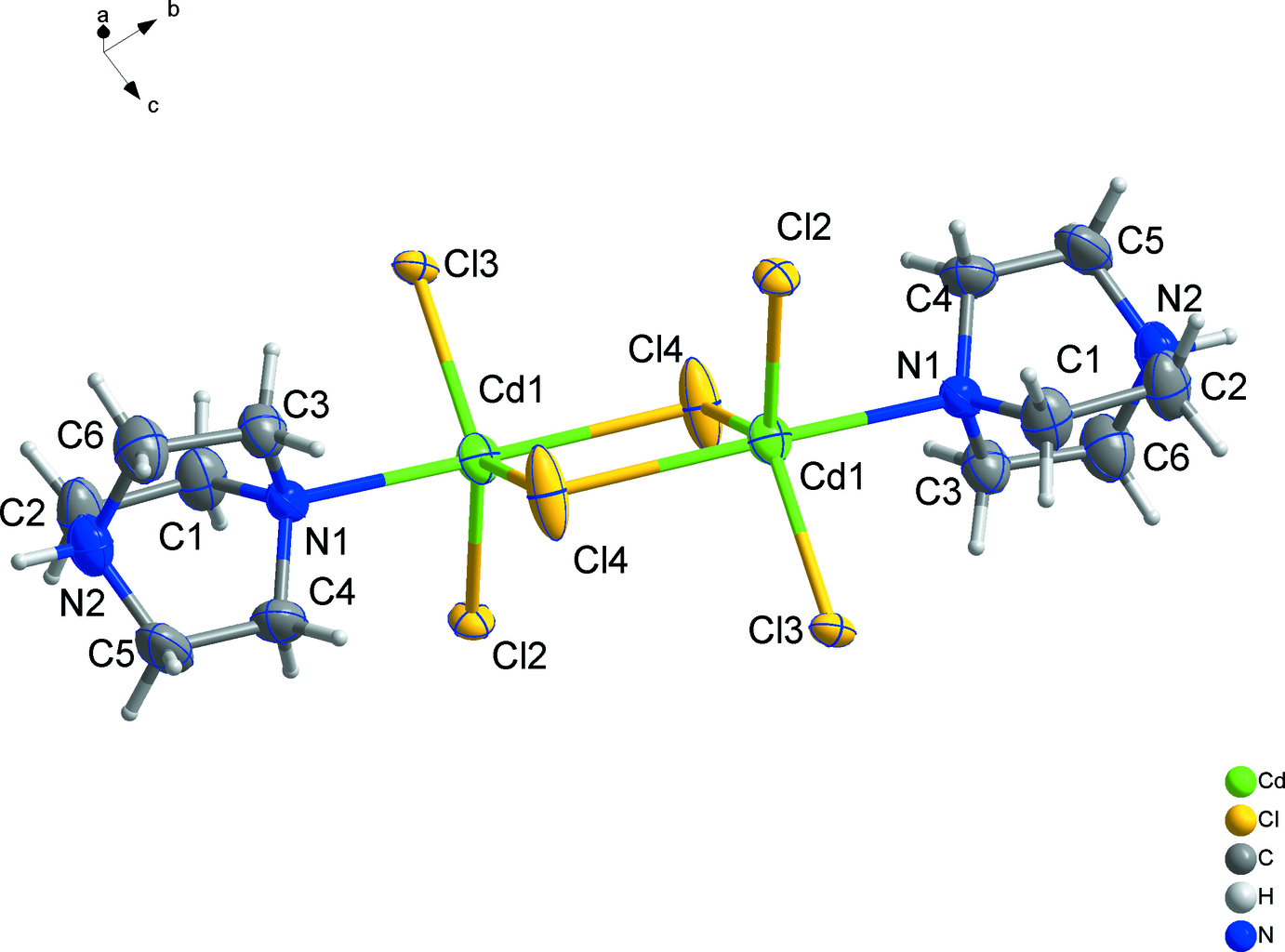

Supplement: Supplementary file 3 [file e-71-0m259-fig1.tif]

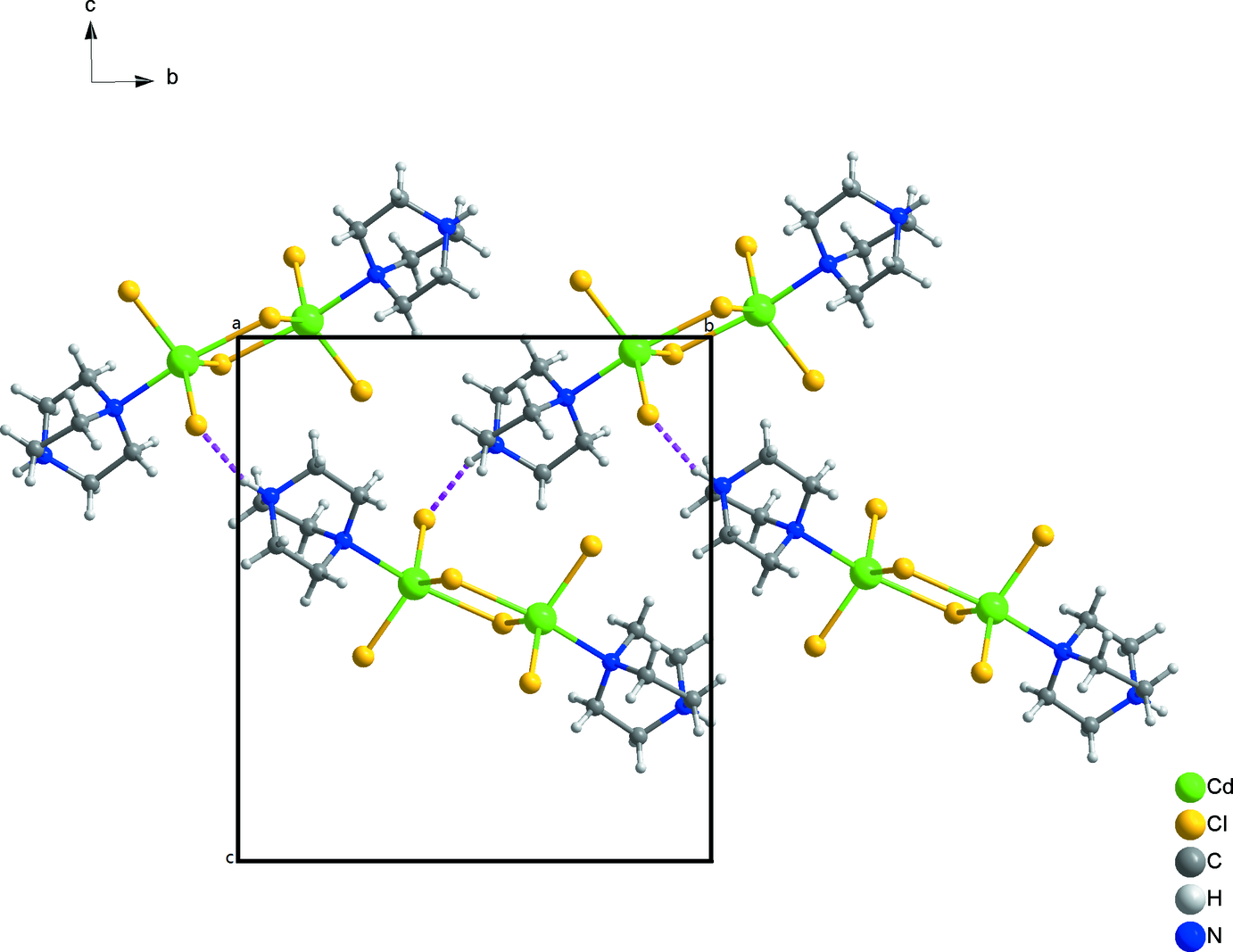

Supplement: Supplementary file 4 [file e-71-0m259-fig2.tif]
